# Diatom-Based Artificial Antigen-Presenting Cells: A Novel Approach for Adaptive Immune Modulation

**DOI:** 10.1021/acsami.5c07766

**Published:** 2025-06-18

**Authors:** Asrizal Abdul Rahman, Isma Liza Mohd Isa, Manus J. Biggs, Abhay Pandit

**Affiliations:** † CÚRAM Research Ireland Centre for Medical Devices, 8799 University of Galway, H91 W2TY Galway, Ireland; ‡ Pharmacology and Therapeutics, School of Medicine, University of Galway, H91 TK33 T Galway, Ireland; § Department of Anatomy, Faculty of Medicine, Universiti Kebangsaan Malaysia, 56000 Kuala Lumpur, Malaysia

**Keywords:** diatom, calcium modification, artificial antigen-presenting cells, T cells, adaptive immunity

## Abstract

Artificial antigen-presenting cells (aAPCs) offer a precise system for modulating immune cells, effectively addressing major challenges in immunotherapy, such as unintended effects. Diatoms have attracted considerable interest as natural templates for biomaterials owing to their surface characteristics, which can replicate those found in cellular structures. In this study, we introduced the creation of calcium-modified diatoms that act as artificial antigen-presenting cells. This innovative strategy aims to enhance immunological interactions and emulate the functions of natural antigen-presenting cells. Our findings indicate that amine polymerization on calcium-modified diatoms improved the attachment of immunomodulatory proteins (anti-CD28 and anti-CD3) to the diatom surface, thereby promoting specific antibody–antigen interactions with human T cells, as evidenced by the formation of immunological synapses, which initiate targeted immune responses. Through costimulatory signaling, we determined robust expression of T cell activation markers, not only during early activation (CD69) but also sustained at a later time point (CD25), associated with increased T cell proliferation. Metabolically, diatom-based aAPCs promote glycolysis over mitochondrial oxidative respiration to meet the elevated energy demand for immune activation and homeostasis. Our findings suggest that the immunofunctionalization of calcium-modified diatoms offers a promising strategy for the development of bioinspired, functional aAPC, which are adaptive immunomodulatory systems, and hold future potential as immunotherapeutic platforms for diseases such as cancer and autoimmunity.

## Graphical abstract

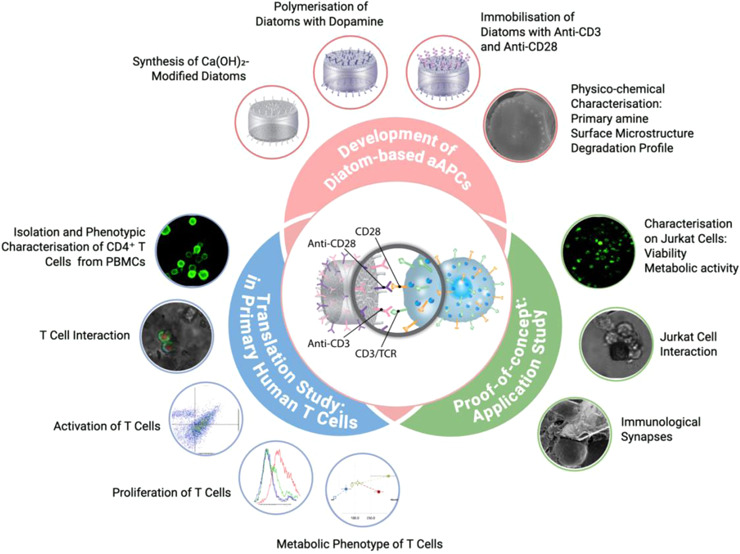

## Introduction

The modulation of the human immune system mainly relies on T cells, which are key components of adaptive immunity that specifically target malignant or infected cells. For T cells to initiate an immune response, they require activation through interaction with antigen-presenting cells (APCs), which present antigenic peptides alongside costimulatory signals. Natural APCs, such as dendritic cells, play a pivotal role in this process; however, difficulties in isolation and manipulation limit their scalability and translation into clinical setting.^1^ Consequently, there is significant interest in developing artificial antigen-presenting cells (aAPCs) that mimic the functions of natural APC, providing controlled and reproducible systems for immune modulation, especially in cancer immunotherapy, autoimmune disorders, infectious diseases, and vaccine development.^2^

A novel approach to developing artificial APCs involves the use of diatoms. Diatoms, which are microscopic algae characterized by silica-based cell walls known as frustules, present a unique topography due to their hierarchically structured frustules, extensive surface area, and biocompatibility.^3^ These attributes render them ideal platforms for cell-mimicking applications.^4^ Our previous studies have shown that diatoms can be incorporated with titanium dioxide (TiO_2_) using titanium­(IV) bis­(ammonium lactato)-dihydroxide (TiBALDH) as a precursor.^5^ Silica diatoms can also be modified by incorporating inorganic elements such as calcium,^6^ aluminum,^7^ zinc,^8^ cadmium^9^ and iron.^10^ Specifically, in calcium species, multiple biomineralization processes, including the formation of silica-chitin-aragonite biocomposites (in the form of calcium carbonate), can coexist through simultaneous silicification and calcification,.^11^ Incorporating calcium into diatoms offers advantages over silica as it optimizes the surface properties of diatoms, allowing the polymerization of the diatom structure for antibody attachment to further facilitate cell signaling processes^12^ and enhance the activation of T cells through antigen–antibody binding.^13^ In this investigation, the application of dopamine coating was selected as a polymerization method due to its excellent adhesive characteristics and its ability to introduce free amine groups onto the diatom surface. The process of dopamine polymerization establishes a solid foundation for the attachment of specific biomolecules, such as antibodies.^14^

Engineering APCs offers precise control of T cell activation through multiple signals, including MHC-peptide complexes, costimulatory ligands, and the release of cytokines.^1^ Herein, we used two key antibodies, anti-CD3 and anti-CD28, following dopamine coating on calcium-modified diatoms to induce T cell activation through costimulatory signaling. These antibodies are essential for T cell activation, as CD3 is part of the T cell receptor (TCR) complex, which initiates T cell signaling, whereas CD28 provides the necessary costimulatory signal for full activation. The combination of these signals is critical for efficient T cell activation and proliferation.^15^ We recently demonstrated that calcium hydroxide (Ca­(OH)_2_) can be taken up into the exoskeleton of diatoms while maintaining their microstructure, providing surface functionalization for antibody-specific binding site application.^16^ By functionalizing dopamine-coated calcium-modified diatoms with CD3 and CD28 antibodies, we aimed to create a robust platform capable of interacting with T cells and facilitating immunological synapse formation for cell activation.

We hypothesized that the immunofunctionalization of dopamine-coated Ca­(OH)_2_-modified diatoms could emulate artificial antigen-presenting cells (aAPCs) by enhancing T cell activation through the costimulatory signaling of CD3 and CD28. This process modulates proliferation and glycolysis while maintaining mitochondrial respiration to ensure metabolic homeostasis. This biomimetic strategy offers a robust platform for the development of next generation immunotherapeutic agents by leveraging both the structural benefits of calcium-modified diatoms and immunological functionalization. It facilitates the controlled costimulatory signaling of aAPCs with antigens presented to T cells, thereby augmenting immune system responses in a targeted and regulated manner.

## Results

This study describes the synthesis of functional diatom-based aAPCs through the oxidative polymerization of Ca­(OH)_2_-modified diatoms with dopamine, forming a layer of polydopamine that provides catechol and amine groups for antibody immobilization with anti-CD3 and anti-CD28 on the diatom surface (Figure 1a). The anti-CD3 and anti-CD28 antibodies presented on the diatom-based aAPCs are specifically recognized by CD3 and CD28 antigens on T cells, initiating their interaction and subsequent full activation (Figure 1b). We evaluated the functionality of diatom-based aAPCs on T cells, focusing on Jurkat cells as a proof-of-concept model and primary human CD4^+^ T cells for translational validation, informing their cellular interactions, activation, proliferation, and energy metabolism via mitochondrial oxidative respiration and glycolysis.

**Figure 1:**
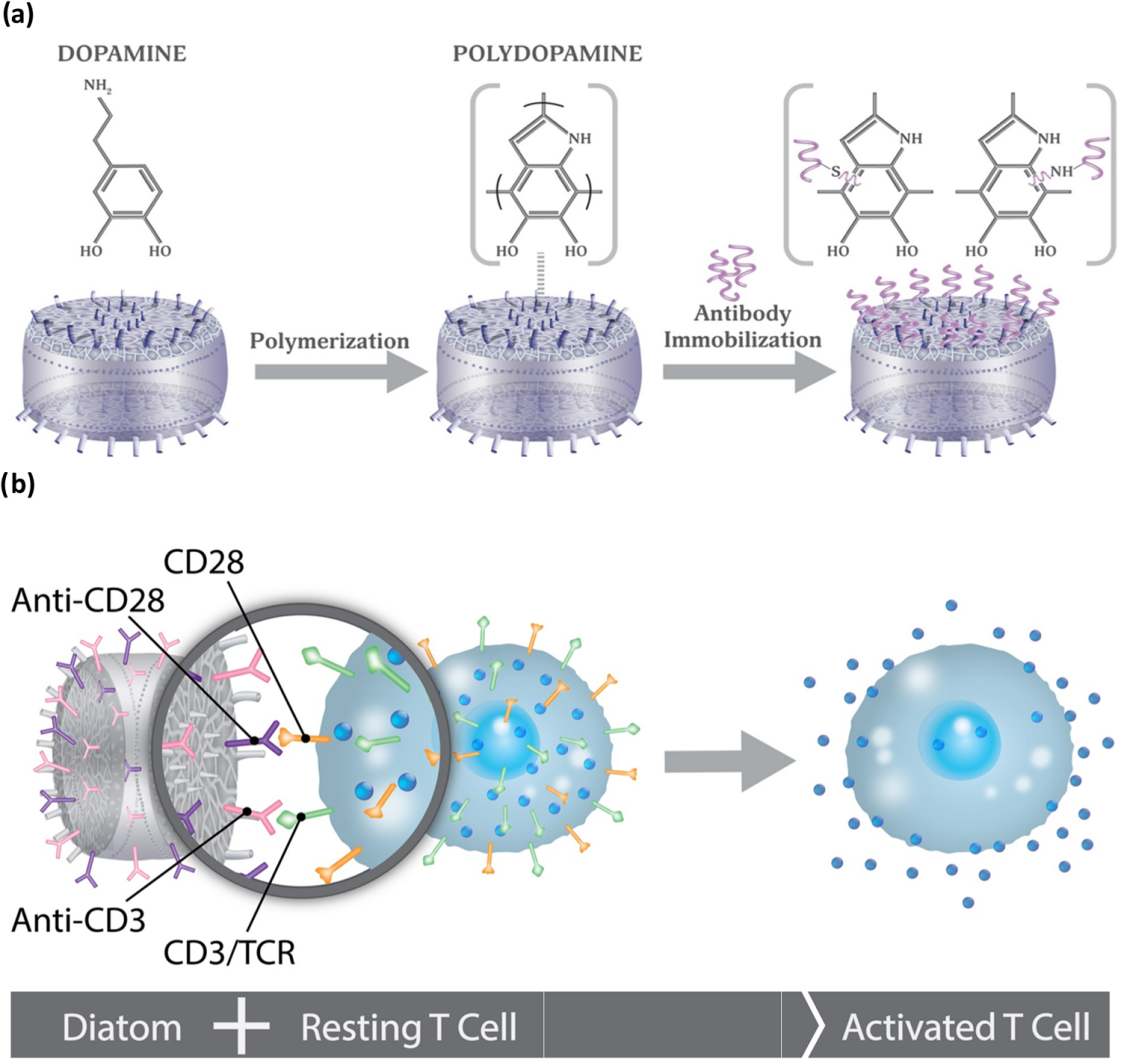
Schematic representations of the fundamental chemical steps for the synthesis of artificial antigen presenting cell (aAPC) using Ca­(OH)_2_-modified diatom. (a) Oxidative polymerization of Ca­(OH)_2_-modified diatoms with dopamine, forming polydopamine. The catechol and primary amine groups of polydopamine react with the amine and thiol groups of the antibodies for further antibody immobilization. (b) Immunofunctionalization of dopamine-coated Ca­(OH)_2_-modified diatoms with antibodies (anti-CD3 and anti-CD28). Anti-CD3 and anti-CD28 presented on diatoms will be recognized by receptors the T cells, leading to their activation.

### Polymerization of Ca­(OH)_2_-Modified Diatoms with Dopamine

We characterized dopamine polymerization of Ca­(OH)_2_-modified diatoms by assessing amine concentration, primary amine groups, degradability, and viability using Fourier transform infrared spectroscopy (FTIR) and scanning electron microscopy (SEM).

The amine functionalization of Ca­(OH)_2_-modified diatoms was conducted using dopamine coating by varying weight-to-weight (w/w) the diatom-to-dopamine ratios (1:1, 1:2, and 1:3) and incubation times (1, 2, 8, and 12 h). The efficiency of amine group attachment was confirmed using the 2,4,6-Trinitrobenzenesulfonic Acid (TNBSA) assay, a standard method for quantifying primary amine groups on diatom surfaces. Among the different ratios tested, the 1:2 diatom-to-dopamine ratio reached a plateau of higher amine functionalization, suggesting an optimal balance between coating thickness and functional group availability (Figure 2a). The 1:2 diatom-to-dopamine ratio was further analyzed for the amine group at 1, 2, 8, and 12 h. We observed that the amine concentration increased over time, indicating steady amine functionalization in dopamine-coated Ca­(OH)_2_ diatoms. A longer exposure to dopamine (8 h) led to higher amine functionalization of Ca­(OH)_2_ diatoms (Figure 2b). FTIR analysis indicated the presence of a primary amine group (−NH_2_) at peaks 3300 and 1650 cm^–1^ in the dopamine-coated Ca­(OH)_2_-modified diatoms, confirming amine functionalization in diatoms (Figure 2c).

**Figure 2:**
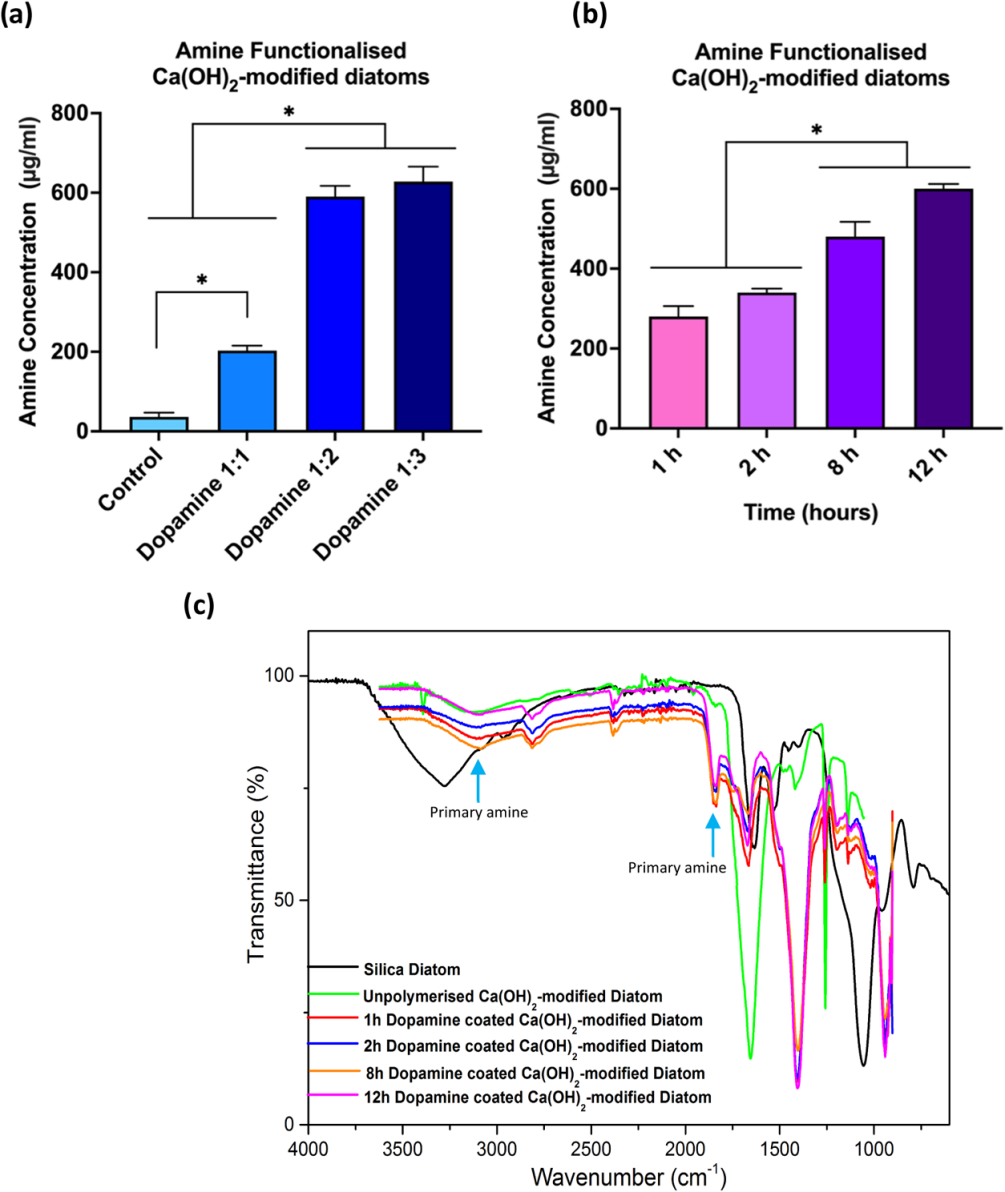
Chemical characterization following polymerization of Ca­(OH)_2_-modified diatoms with dopamine. (a) A trend of increasing concentration of amine groups was observed as the diatom-to-dopamine concentration ratio increased. The plateau in the 1:2 group indicates the maximum capacity of amine groups, reflecting the efficiency of amine functionalization. Therefore, the 1:2 diatom-to-dopamine ratio was chosen for subsequent analysis. (b) A temporal increase in amine concentration was evident, with a plateau observed at 8 h, indicating stable amine functionalization. (c) FTIR analysis showed the presence of primary amine groups (−NH_2_) in the dopamine-coated Ca­(OH)_2_-modified diatoms, with peaks at 3300 and 1650 cm^–1^, informing the introduction of amine and catechol-related peaks after dopamine polymerization, while maintaining baseline silanol bands. Data are presented as means ± SEM *p < 0.05; *n* = 3. One-way ANOVA was used for (a, b).

SEM analysis showed that the surface fultoportulae microstructure of the dopamine-coated Ca­(OH)_2_-modified diatoms was retained in both the central and peripheral regions, indicating that the microstructure of the Ca­(OH)_2_-modified diatoms remained unaltered during the oxidative polymerization by dopamine (Figure 3a). Interestingly, the calcium-modified diatoms exhibited stable dopamine coatings with minimal degradation over time, most likely because of the role of calcium in stabilizing the polydopamine layer (Figure 3b). Fluorescence microscopy of the LIVE/DEAD assay indicated the presence of a strong green signal, suggesting higher viability of Jurkat cells, which were round and dispersed in both dopamine-coated and uncoated Ca­(OH)_2_-modified diatom cultures, comparable to the Jurkat cell control. In the silica diatom group, a green signal was observed, indicating viable Jurkat cells. However, there was minimal red fluorescence, and some cells exhibited disintegration-shaped cells, suggesting necrotic cells (Figure 4a). Using the alamarBlue assay, we demonstrated significantly higher metabolic activity in Jurkat cells in the presence of dopamine-coated Ca­(OH)_2_-modified diatoms than in all other groups on days 1, 2, and 7, indicating no cytotoxic effects on cells (Figure 4b).

**Figure 3:**
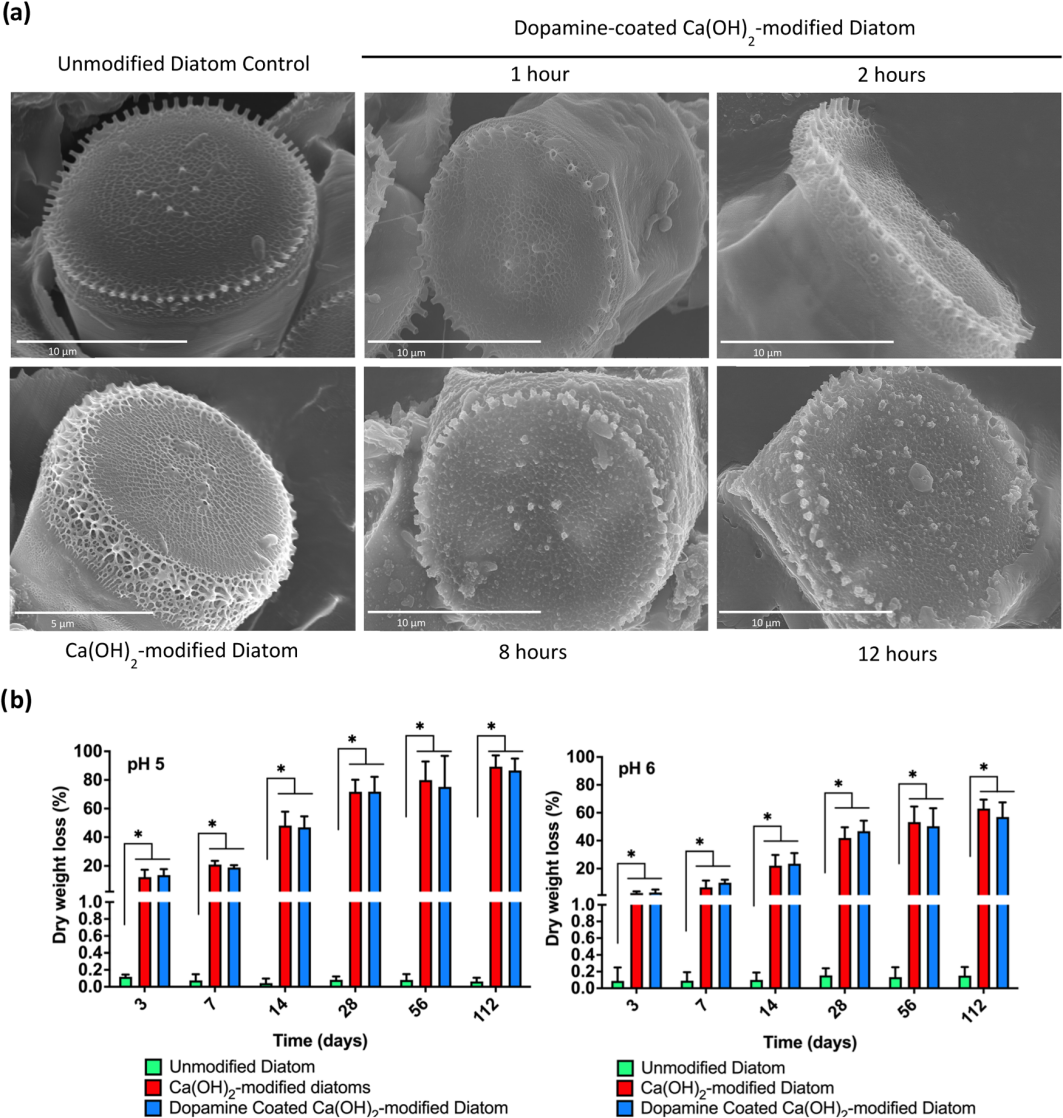
Characterization of amine-functionalized Ca­(OH)_2_-modified diatoms for surface morphology and degradation. (a) SEM analysis revealed surface fultoportulae microstructure of Ca­(OH)_2_-modified diatoms remained intact following dopamine-mediated oxidative polymerization for up to 12 h. (b) Minimal degradation profile of Ca­(OH)_2_-modified diatoms in comparison to unmodified diatoms in aqueous condition. Data are presented as means ± SEM *p < 0.05; *n* = 3. Two-way ANOVA was used for (b). Scale bars represent 10 μm for (a) unmodified diatom control and Dopamine-coated Ca­(OH)_2_-modified diatom, and 5 μm for (a) unpolymerized Ca­(OH)­2-modified diatom.

**Figure 4:**
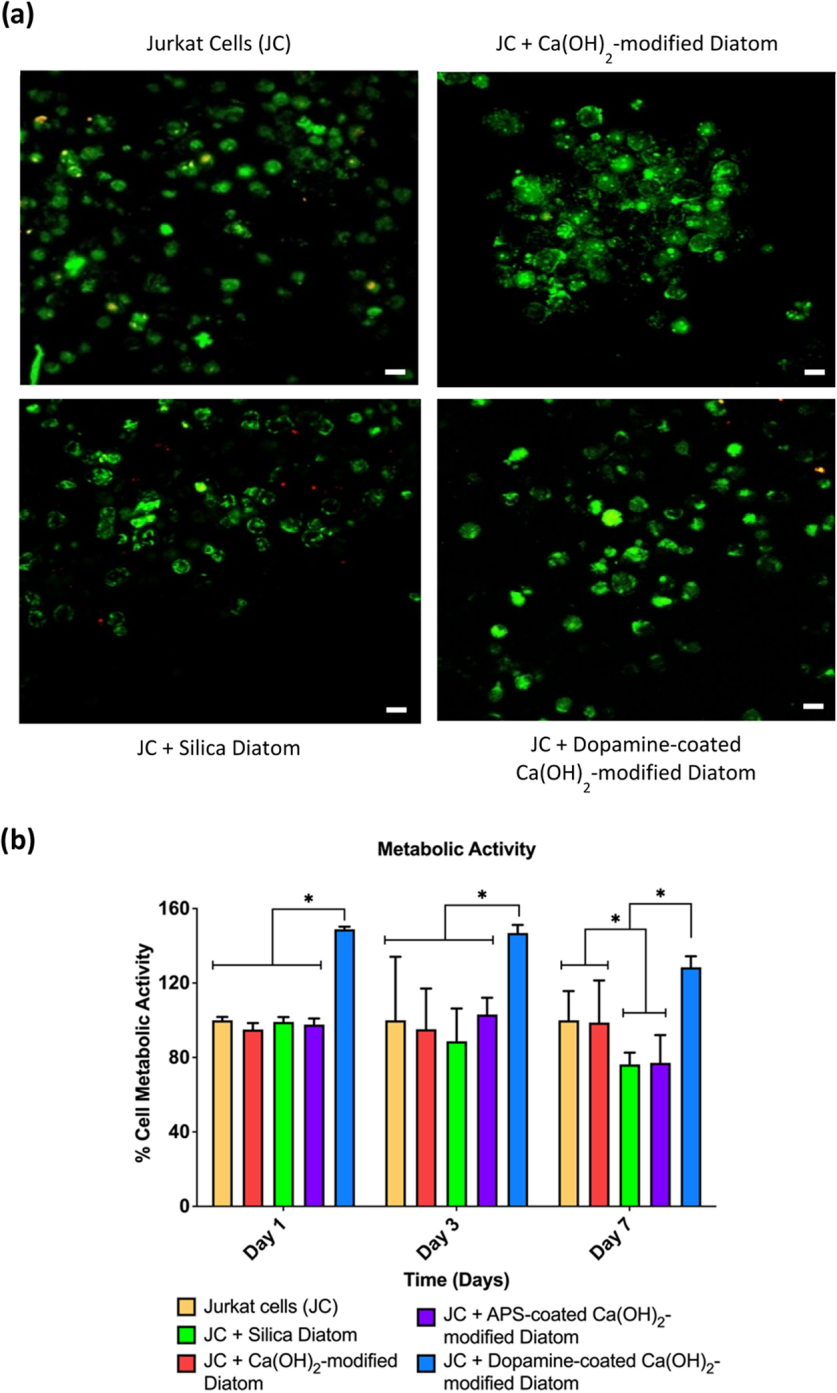
Viability of Jurkat cells following treatment with dopamine-coated Ca­(OH)_2_-modified diatoms. (a) Robust viability of Jurkat cells was observed in the presence of dopamine-coated Ca­(OH)_2_-modified diatoms at day 5. (b) Metabolic activity was significantly higher in dopamine-coated Ca­(OH)_2_-modified diatoms compared to other groups, indicating no evidence of cytotoxicity. Data are presented as means ± SEM *p < 0.05; *n* = 3. Two-way ANOVA was used for (b). Scale bars represent 10 μm for (a).

### Antibody Immobilization on Dopamine-Coated Ca­(OH)_2_-Modified Diatoms

Immunofunctionalization of dopamine-coated Ca­(OH)_2_-modified diatoms with anti-CD3 and anti-CD28 was confirmed using fluorescence microscopy, SEM, and cell density measurements.

The successful immobilization of anti-CD3 and anti-CD28 antibodies on the dopamine-coated calcium-modified diatom surfaces was confirmed through fluorescence microscopy using fluorophore-conjugated antibodies, PE (red label) and FITC (green label) for anti-CD3 and anti-CD28, respectively (Figure 5). These fluorescent labels allowed the visualization and quantification of antibody attachment on the diatom surface, revealing uniform and stable antibody attachment across the coated diatoms.

**Figure 5:**
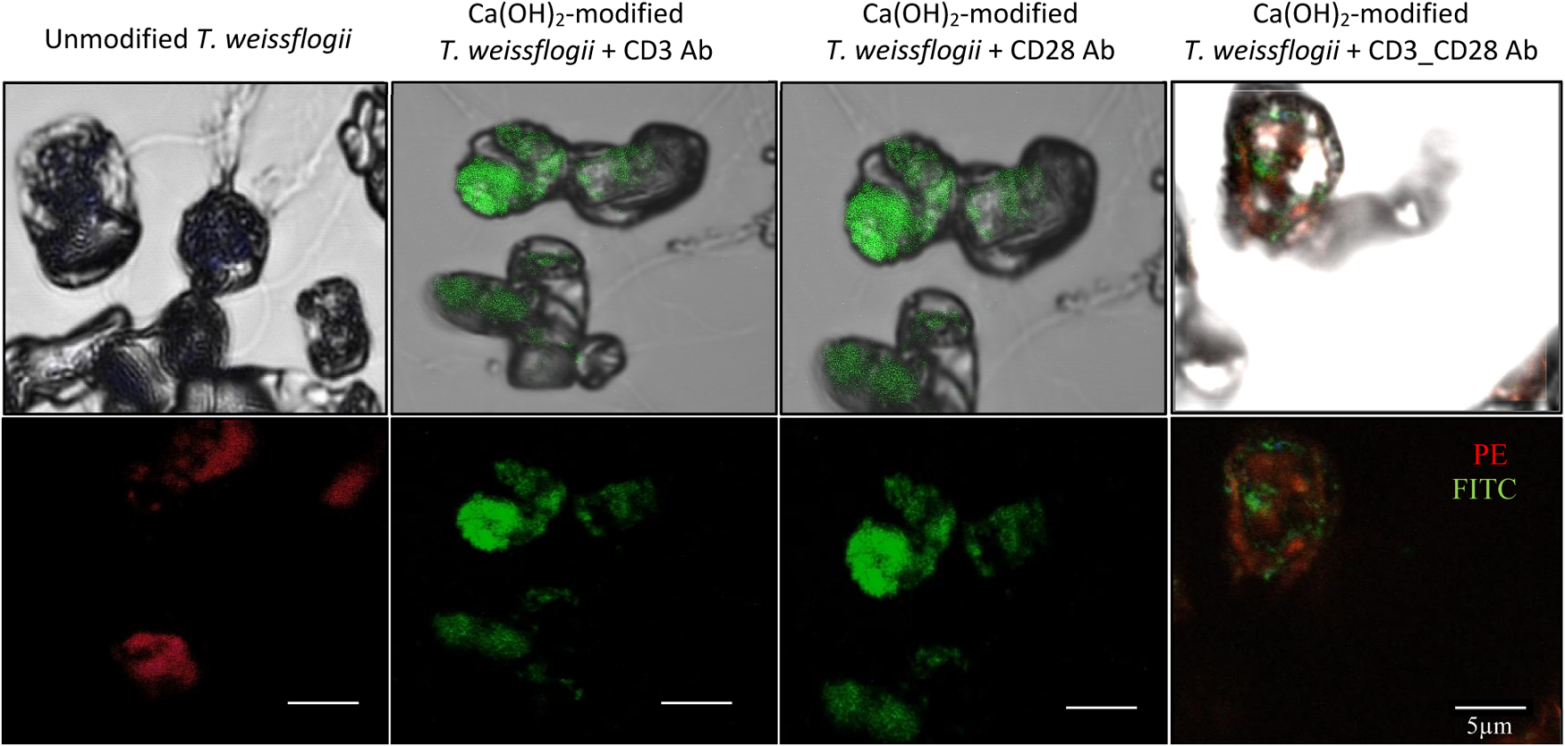
Fluorescence microscopy images revealed the immobilization of PE-labeled anti-CD3 (red) and FITC-labeled anti-CD28 (green) antibodies on the surface of Ca­(OH)_2_-modified diatoms following surface functionalization with a polydopamine coating. Scale bars represent 5 μm. Adapted images from Rahman et al.^16^

SEM analysis revealed the presence of protein aggregates of antibodies on the diatom surface. The surface microstructure of anti-CD3_anti-CD28 functionalized dopamine-coated Ca­(OH)_2_-modified diatoms retained their unique fultoportulae feature in both the central and peripheral regions. This suggests that the microstructure of the Ca­(OH)_2_-modified diatoms was not altered during antibody immobilization (Figure 6a).

**Figure 6:**
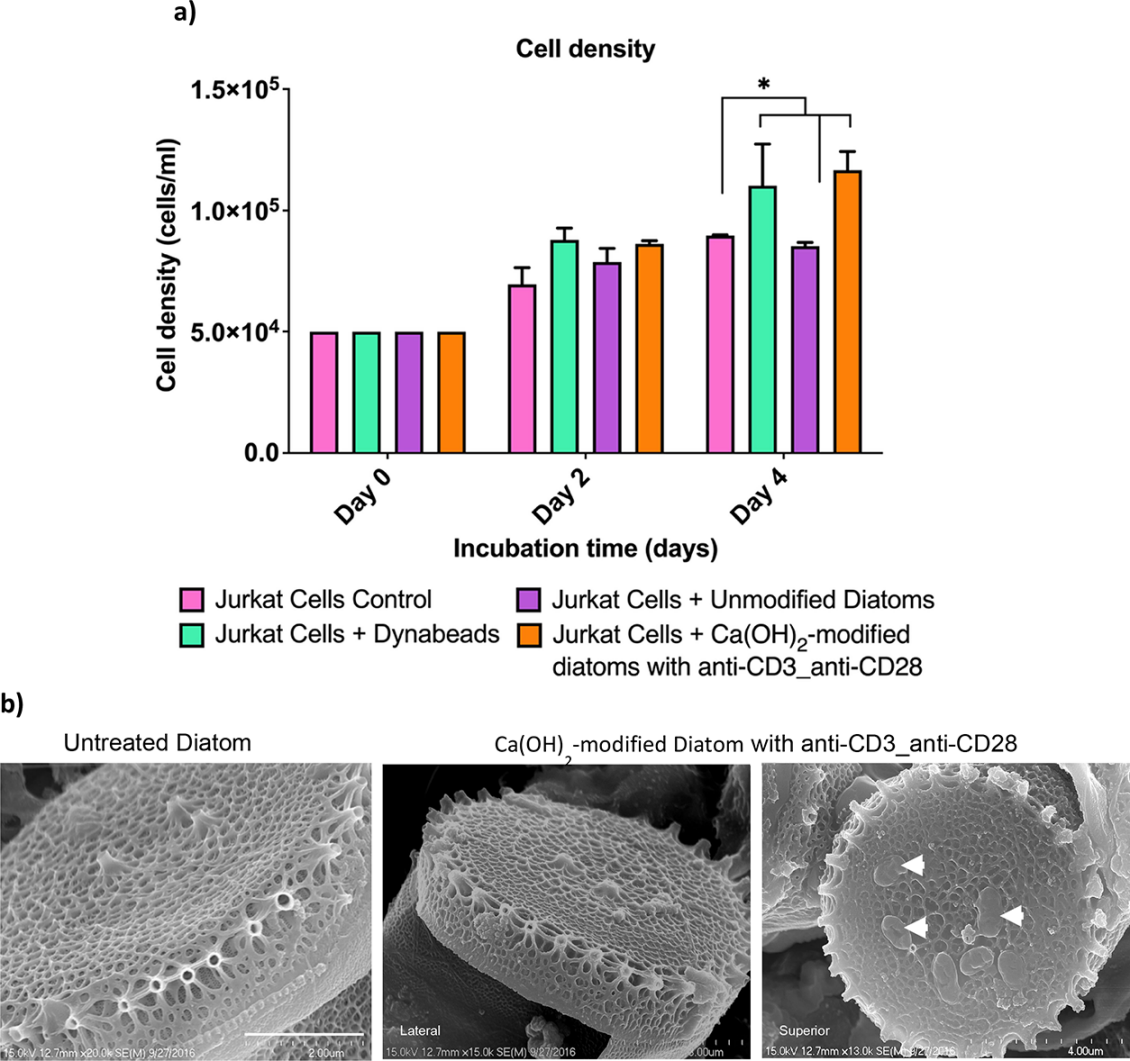
Characterization of anti-CD3_anti-CD28 immobilized amine functionalized-Ca­(OH)_2_-modified diatoms for Jurkat cell density and surface morphology. (a) A higher density of Jurkat cells was observed when treated with these diatoms by day 4. (b) SEM images indicated the presence of protein aggregates of antibodies (marked in white arrows) on Ca­(OH)_2_-modified diatoms compared to control silica-based unmodified diatoms. Data are presented as means ± SEM *p < 0.05; *n* = 3. Two-way ANOVA was used for (a). Scale bars represent 2 μm for (b).

### Jurkat Cell Interaction with Diatom-Based aAPC

The interaction and activation between Jurkat cells and diatom-based aAPCs were determined in the study of cell density, binding affinity by time-lapse light microscopy, percentage of interaction, and SEM analysis for evidence of immunological synapse.

Diatom-based artificial APC were subjected to incubation with Jurkat cells for a period extending up to 4 days. On the second day, all groups exhibited an increase in cell density when compared to the initial baseline. Our results indicate a marked enhancement in the cell density of Jurkat cells within the group treated with anti-CD3_anti-CD28 functionalized dopamine-coated Ca­(OH)_2_-modified diatoms, achieving levels comparable to those observed in the Dyna Bead group, which serves as the established standard. This finding underscores the efficacy of diatom-based aAPCs in facilitating cell proliferation (Figure 6b).

We observed there was no visible interaction between Jurkat cells and unmodified diatoms (control), in contrast, we indicated that active interaction between Jurkat cells and Dyna Beads. Similarly, when Jurkat cells were exposed to anti-CD3_anti-CD28 functionalized dopamine-coated Ca­(OH)_2_-modified diatoms, they showed robust interaction, as observed by light microscopy, suggesting that Jurkat cells recognize and interact with diatoms (Figure 7a). A significant increase in the percentage of interactions in Jurkat cells was observed in the presence of anti-CD3_anti-CD28 functionalized dopamine-coated Ca­(OH)_2_-modified diatoms, suggesting higher affinity binding through the TCR and diatom-based aAPCs, thus forming stable immunological synapses between them (Figure 7b). Time-lapse microscopy revealed specific interactions between Jurkat cells and diatom-based aAPCs (Supporting Video 1). High-resolution SEM analysis confirmed the surface formation of immunological synapses between Jurkat cells and diatom-based aAPCs anti-CD3_anti-CD28 functionalized dopamine-coated Ca­(OH)_2_-modified diatoms (Figure 7c). Our findings demonstrate that dopamine-coated calcium-modified diatoms not only facilitate stable antibody attachment but also promote the viability and interaction of Jurkat cells with the diatom surface.

**Figure 7:**
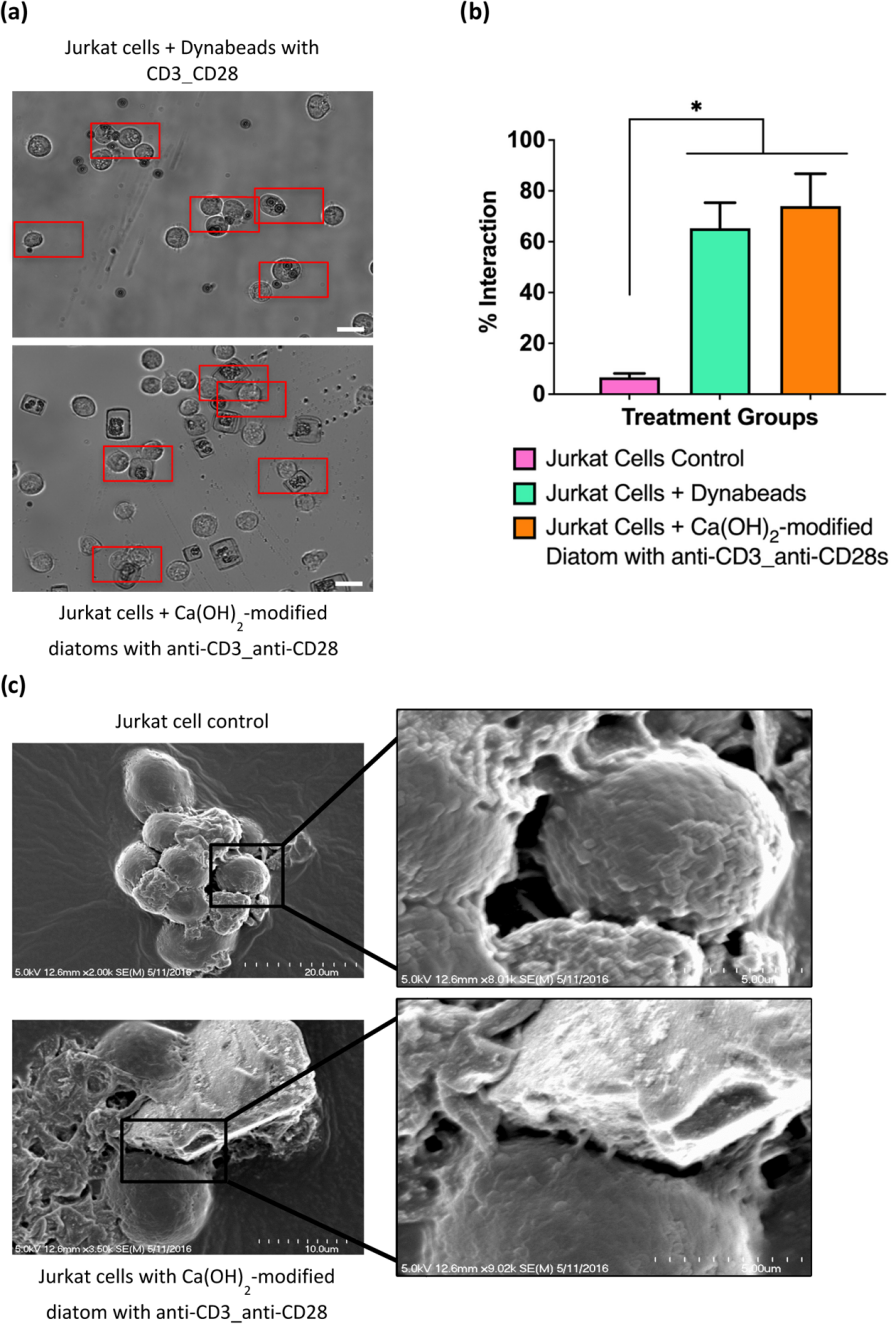
Interaction of Jurkat cells and anti-CD3_anti-CD28 immobilized amine-functionalized Ca­(OH)_2_-modified diatoms (diatom-based aAPCs). (a) Jurkat cells exposed to these diatoms demonstrated robust binding, as observed in light microscopy. (b) Higher interaction of Jurkat cells with these diatoms were evident when compared to control unmodified diatoms. (c) SEM images revealed interactions between Jurkat cells and anti-CD3_anti-CD28-treated diatoms, forming stable immunological synapses. Data are presented as means ± SEM *p < 0.05; *n* = 3. One-way ANOVA was used for (b). Scale bars represent 20 μm for (a) and 10–20 μm for (c).

### Activation of Human Primary CD4^+^ T Cells Following Interaction with Diatom-Based aAPCs

We assessed the activation of primary T cells (CD4^+^ peripheral blood mononuclear cells, PBMCs) following their interaction with diatom-derived aAPCs using flow cytometry and molecular analyses.

PBMCs were isolated using density gradient centrifugation. A distinct PBMC layer was clearly present, indicating effective density gradient separation (Supporting Figure S1a). Phenotypically, flow cytometry-based magnetic sorting confirmed that the high CD4^+^-expressing population (rightmost cluster) represented CD4^+^ T cells, suggesting the purity of untouched CD4^+^ PBMCs and highlighting the presence of primary T cells (Supporting Figure S1b). A single dominant population appears in the lower forward scatter and side scatter, indicating a homogeneous CD4^+^ T cell population (Supporting Figure S1c). The CD4^+^ PBMCs were labeled with Fluo-4 AM, a calcium-sensitive dye that indicates intracellular calcium levels. Using fluorescence microscopy, we determined the rounded morphology and well-distributed Fluo-4-labeled CD4^+^ PBMCs at 2 h postincubation with anti-CD3_anti-CD28 functionalized dopamine-coated Ca­(OH)_2_-modified diatoms, as indicated in green, which suggests viable CD4^+^ T cells and the presence of intracellular calcium signaling at early phase of T cell activation. (Figure 8a). Mean fluorescence intensity (MFI) of Fluo-4 signals in CD4^+^ PBMCs showed a statistically significant increase in the diatom-based aAPC group compared to the untreated control. These results confirm calcium influx consistent with early T cell activation (Figure 8b).

**Figure 8:**
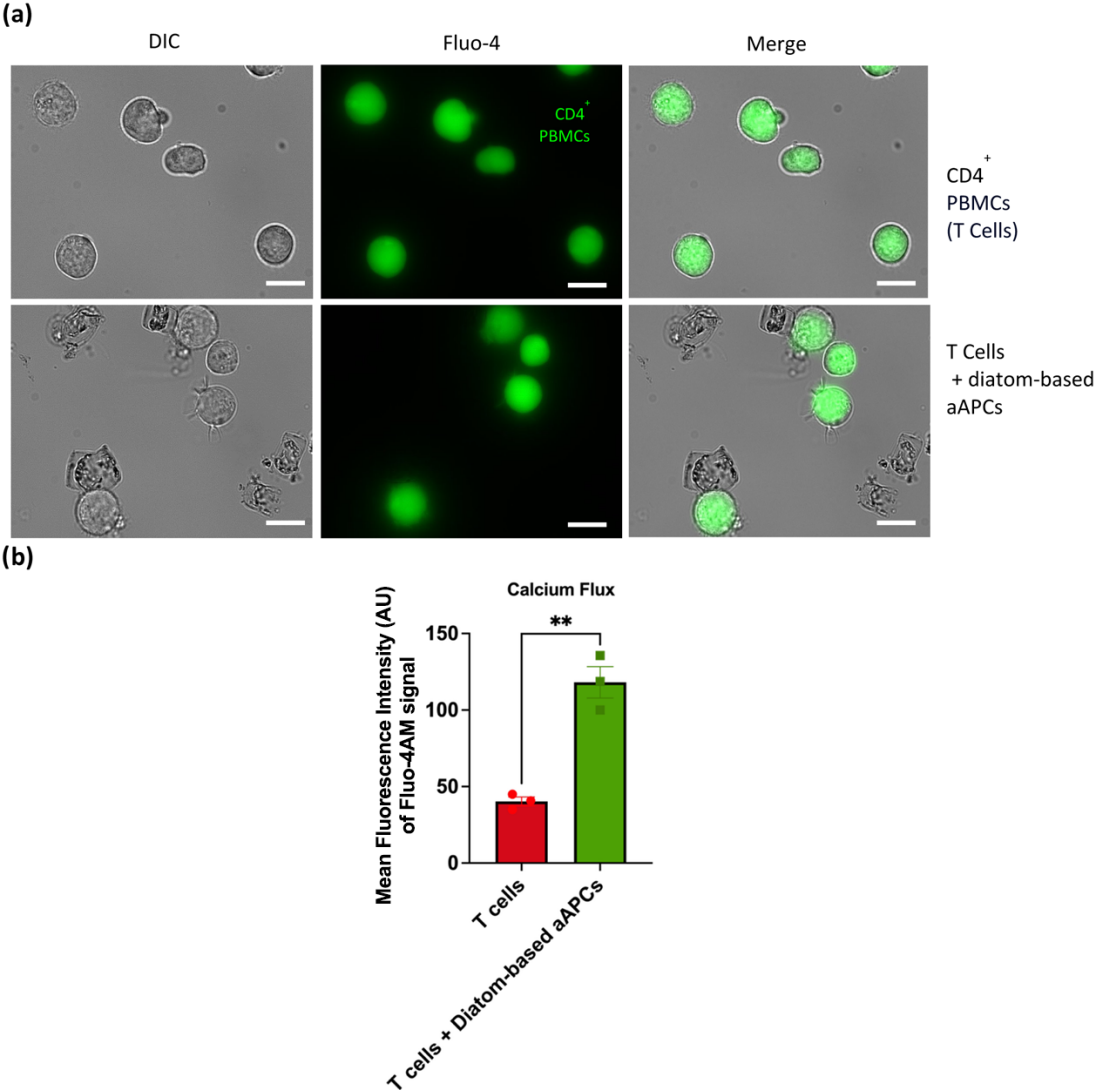
Static Fluo-4 imaging for calcium signaling and T cell activation. (a) Fluorescence microscopy demonstrated influx of calcium in fluo-4 labeled-CD4^+^ PBMCs at 2 h postincubation with aAPCs-derived diatoms. There were evidence of robust binding between them indicated by immunological synapse. (b) A higher mean fluorescent intensity of the calcium signal was observed in activated T cells treated with diatom-based aAPCs compared to untreated T cells. Data are presented as means ± SEM *p < 0.05; *n* = 3. Scale bars represent 10 μm for (a).

Similarly, we observed the presence of intracellular calcium signaling in Fluo-4-labeled CD4^+^ PBMCs (indicated by calcium dye) when cultured with diatoms in calcium feeding medium compared to silica control and calcium starving condition (Figure 9). Ca­(OH)_2_-modified diatoms immobilized with both anti-CD3 and anti-CD28 antibodies exhibited robust T cell binding, with stable synapse formation evident as early as 30 min postincubation. This finding suggests the formation of immunological synapses, a structure that forms following the interaction of antibodies and antigens of T cells, thus initiating signaling cascades for T cell activation.

**Figure 9:**
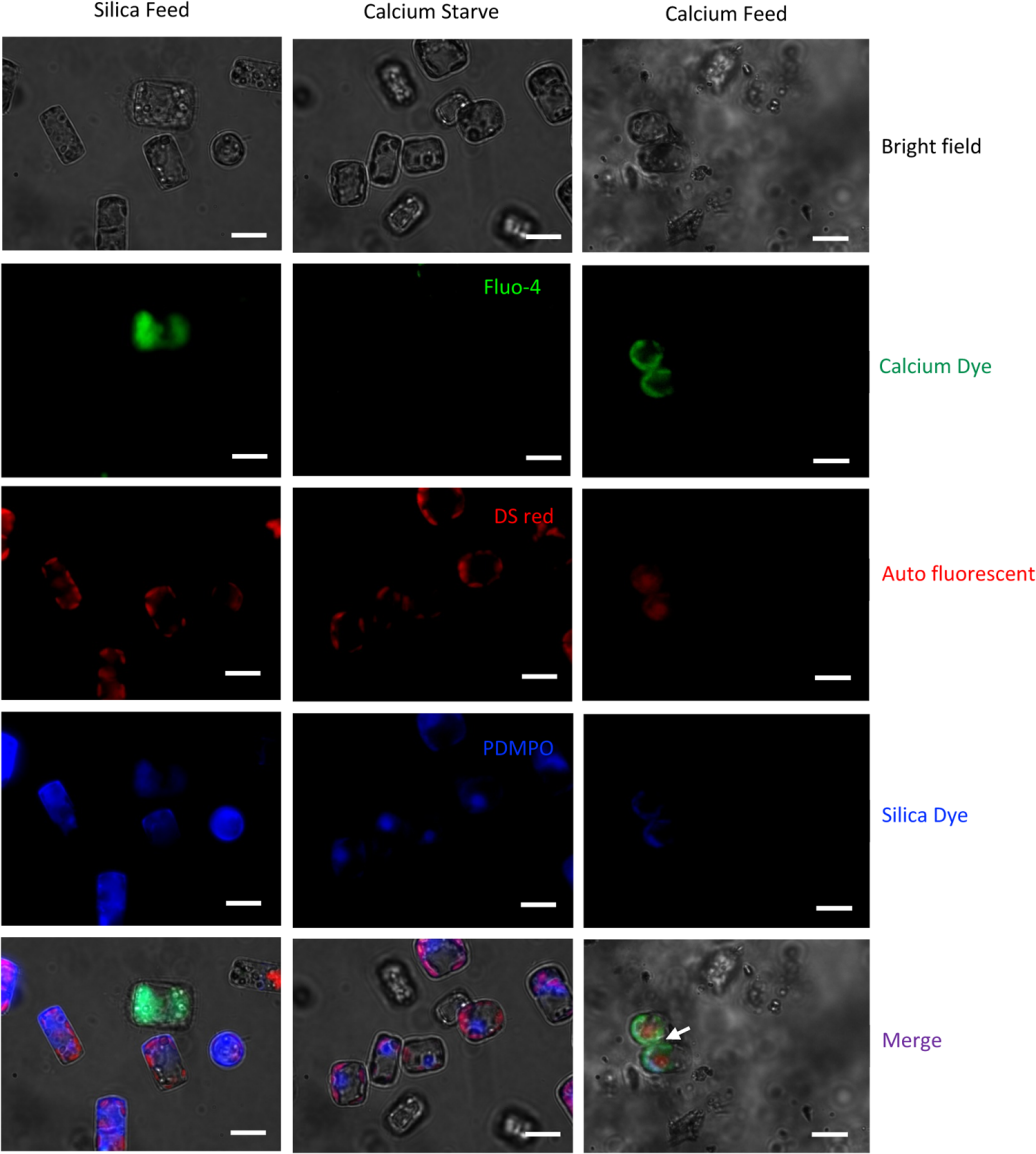
Interaction between primary human T cells (CD4^+^ peripheral blood mononuclear cells (PBMCs)) and aAPCs-derived diatoms in silica-feed, calcium starve and calcium-feed conditions. Evidence of immunological synapse (marked in a white arrow) between CD4^+^ PBMCs and diatom-based aAPCs in calcium feed group in comparison to calcium starving and silica feeding control. *n* = 3. Scale bars represent 10 μm.

To evaluate T cell activation, RT-qPCR was employed to measure the expression of the activation markers CD69 and CD25 on CD4^+^ PBMCs. CD4^+^ PBMCs (T cells) treated with diatom-based aAPCs (anti-CD3_anti-CD28 functionalized dopamine-coated Ca­(OH)_2_-modified diatoms) showed a marked increase in CD69 expression at 10 h, reaching significantly higher levels than the controls (Figure 10ai), indicating the earliest marker upregulated during T cell activation, often appearing within hours of antigen recognition. By 24 h, CD25, the interleukin-2 receptor α chain associated with sustained T cell activation and proliferation, was also upregulated, suggesting that these diatom-derived aAPCs effectively support prolonged activation signaling in T cells (Figure 10aii).

**Figure 10:**
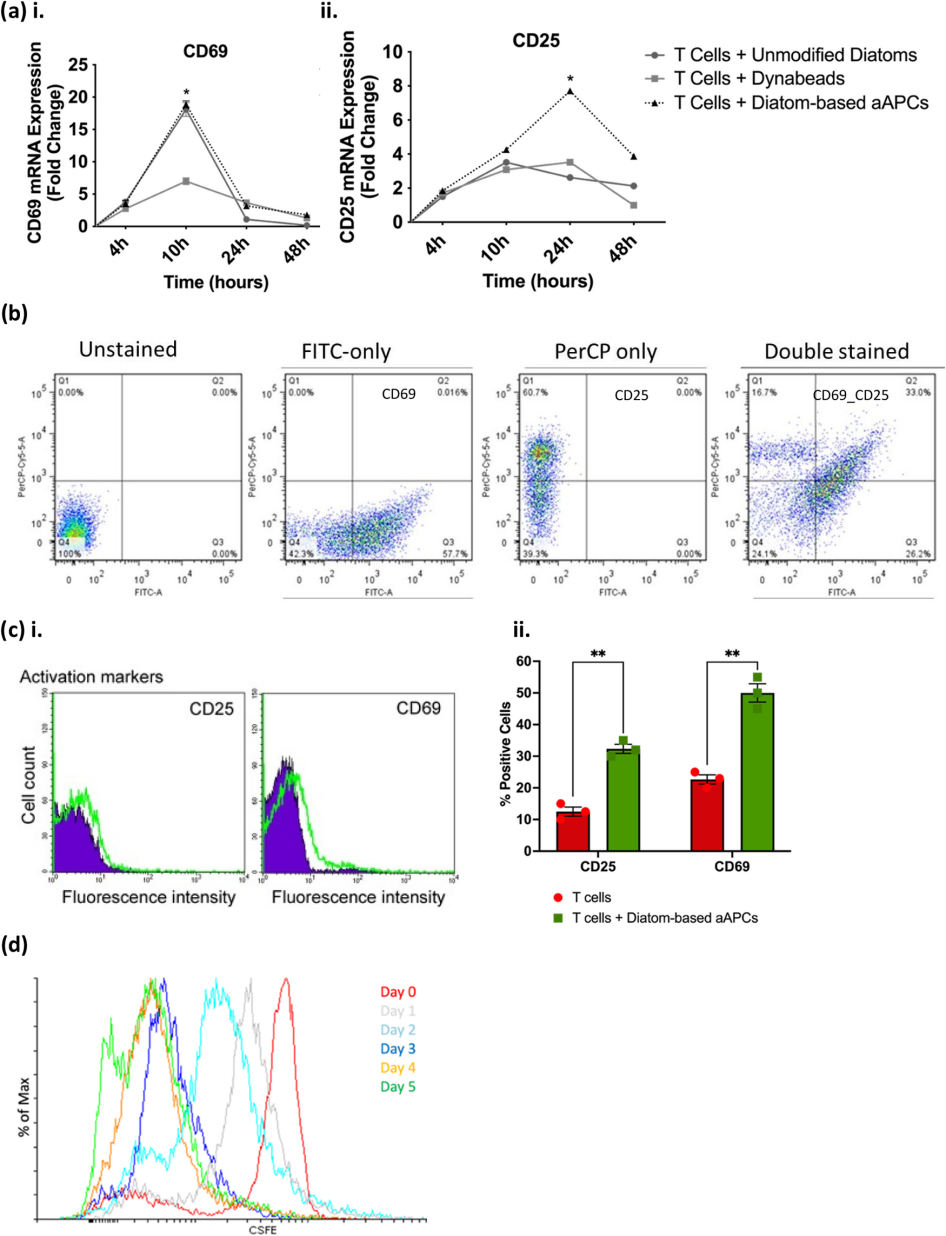
Activation and proliferation of primary T cells (CD4^+^ PBMCs) in the presence of aAPC-derived diatoms. (a) mRNA expression of T cell surface markers showed upregulation of CD69 at an early time point, followed by CD25 at a later time point, indicating the temporal activation of T cells following interaction with aAPC-derived diatoms. (b, cii) Activation of T cell surface markers (CD69 and CD25) were validated by density plots and fluorescent intensity in flow cytometry analysis. (cii) T cells showed a higher count of positively stained cells with CD69 and CD25 when treated with diatom-based aAPCs compared to the untreated control. (d) An overlay of the CFSE signals up to day 5 demonstrated general left-shift signals that reflect a reduction in carboxyfluorescein diacetate succinimidyl ester (CFSE) fluorescence (480/530/30 nm channel), indicating T cell proliferation. Data are presented as means ± S.E.M.; *n* = 3, *p < 0.05, two-way ANOVA and multiple *t* tests were used for (a, cii), respectively.

Flow cytometry analysis indicated density plots Q3 (FITC^+^ PerCP^–^, 57.7%) and Q1 (PerCP^+^ FITC^–^, 60.7%) for the highly positive expression of CD69 and CD25, respectively. The density plot Q2 (PerCP^+^ FITC^+^, 33.0%) demonstrated double-positive cells coexpressing both markers on CD4^+^ PBMCs exposed to anti-CD3_anti-CD28 functionalized dopamine-coated Ca­(OH)_2_-modified diatoms compared to control unmodified diatoms (Figure 10b). This result confirmed T cell activation through a significant increase in CD69 and CD25 expression. Flow cytometry histograms demonstrated a rightward shift in fluorescence intensity in CD4^+^ PBMCs exposed to the anti-CD3_anti-CD28 functionalized dopamine-coated Ca­(OH)_2_-modified diatom group (green label), indicating increased CD25 expression in response to activation compared to the control nonfunctionalized diatom group (purple label), which was indicated by a low fluorescence intensity, suggesting minimal CD25 expression. We observed a similar trend of fluorescence intensity shifts to the right in CD4^+^ PBMCs exposed to the anti-CD3_anti-CD28 functionalized dopamine-coated Ca­(OH)_2_-modified diatom group (green label), indicating higher CD69 expression upon activation. The control group (purple label) showed low fluorescence intensity, indicating that CD69 was minimally expressed in resting cells (Figure 10c).

### Proliferation of Activated Human Primary CD4^+^ T Cells

Using flow cytometry analysis, cell proliferation was assessed in CD4^+^ PBMCs exposed to anti-CD3_anti-CD28 functionalized dopamine-coated Ca­(OH)_2_-modified diatoms over 5 days by tracking carboxyfluorescein diacetate succinimidyl ester (CFSE) dye dilution, a method for monitoring cell division. The flow cytometry histogram plot showed a single peak at a high CFSE intensity on day 0 (red peak), indicating that no cell division had occurred. On days 1 and 2, we observed a slight shift to the left (gray and turquoise peaks), indicating that some cells had started dividing, but proliferation was still limited. However, the CFSE peak shifted further left (navy blue peak) on days 3, 4, and 5, indicating increased proliferation on day 3. Additionally, more heterogeneity appeared in the peaks (yellow and green) on day 5, suggesting different numbers of divisions (Figure 10d).

### Mitochondrial Oxidative Respiration and Glycolysis in Activated Human Primary CD4^+^ T Cells

We determined the functional diatom-based aAPCs in primary T cells (CD4^+^ PBMCs) on energy metabolism, a hallmark of T cell activation, using Seahorse extracellular flux analysis on extracellular acidification rate (ECAR) and oxygen consumption rate (OCR), which are critical indicators of mitochondrial respiratory capacity and glycolysis.

The ECAR assay exhibited a stable baseline reading over time in the control T cell group, confirming that glycolysis is substrate-dependent. Conversely, we observed moderate increases in ECAR in the positive control of DynaBead-activated T cells at the early time point of 30 min, which subsequently plateaued until 150 min, indicating a potential saturation of glycolytic activity. Diatom-based aAPC-activated T cells exhibited a progressive increase in ECAR over time, peaking at 110 min, which indicated the highest glycolytic activity relative to control T cells and DynaBead-activated T cells, suggesting robust respiration via the glycolytic pathway during T cell activation (Figure 11a).

**Figure 11:**
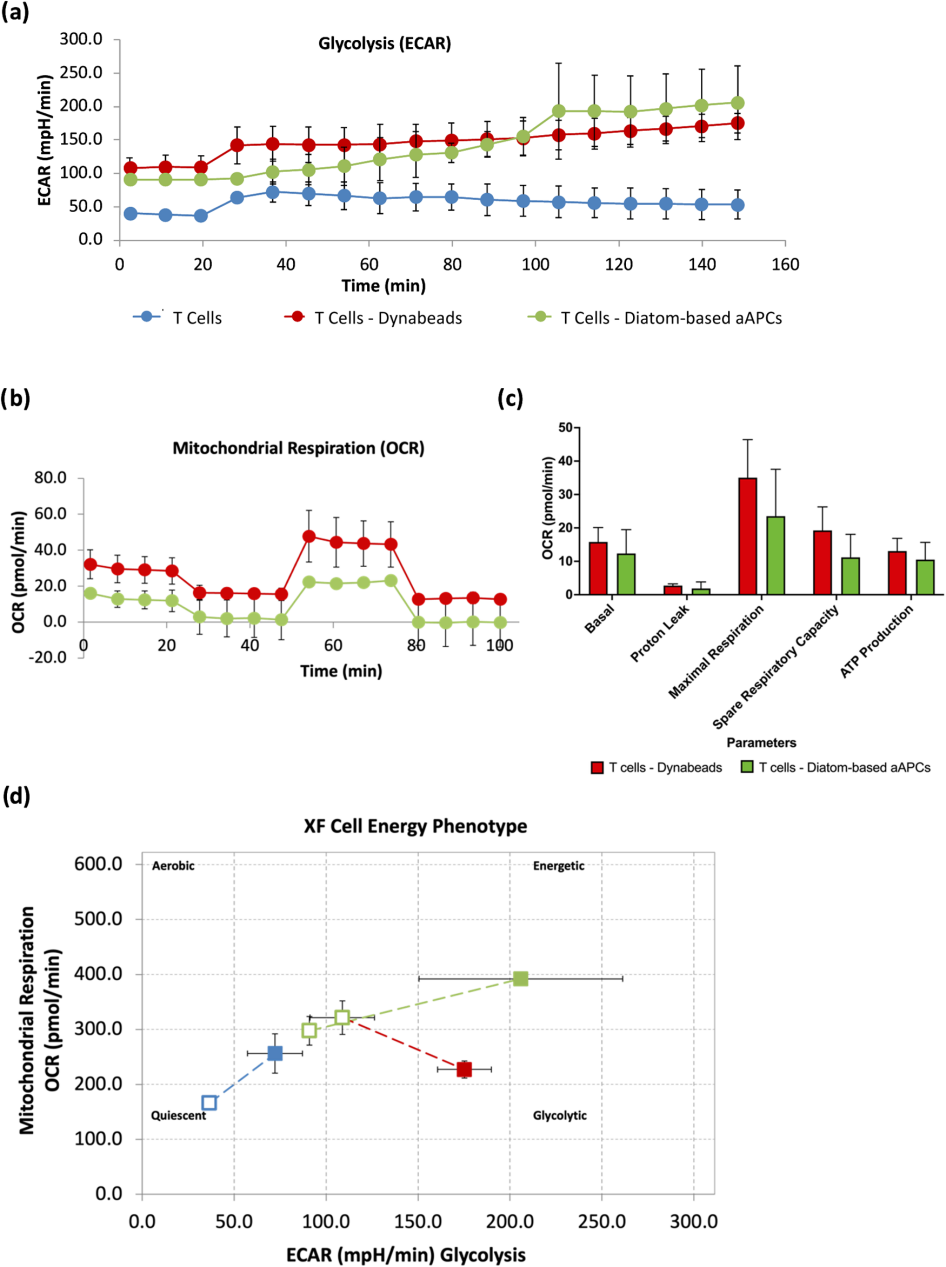
Mitochondrial respiratory capacity of primary human T cells (CD4^+^ PBMCs) in the presence of aAPC-derived diatoms by Seahorse XF cell mito stress test. (a) The extracellular acidification rate (ECAR) revealed an increasing trend in glycolysis in activated T cell–diatom group. (b) Continuous O_2_ consumption (OCR) in activated T cell–diatom group was comparable to that of activated T cell–DynaBead group. (c) No significant differences in mean OCR were observed for mitochondrial respiratory parameters in T cells in the presence of aAPC-derived diatoms or DynaBeads. (d) Activated T cell–diatom cultures demonstrated a cell energy phenotype of mitochondrial respiration, progressing toward glycolysis phase while maintaining mitochondrial respiration. Data are presented as means ± SEM *p < 0.05; *n* = 3. Multiple unpaired *t* tests were performed.

T cells exposed to Dyna Beads exhibited elevated basal and maximal OCR following FCCP treatment, a strong uncoupler of mitochondrial oxidative phosphorylation. Conversely, there was a slight decrease in OCR after oligomycin (indicating a decline in ATP production) and rotenone treatment, confirming functional ATP-linked respiration and mitochondrial specificity, respectively. Although OCR measurements were slightly lower in activated T cells stimulated by diatom-based aAPCs, the overall OCR pattern was similar to that of the T cell–Dyna Bead group (Figure 11b). In particular, there were no significant differences in basal and maximal respiration, proton leak, spare respiratory capacity, or ATP generation between the two groups (Figure 11c). T cells exposed to diatom-based aAPCs demonstrated active mitochondrial respiratory phenotype, which gradually transitioned to glycolysis to meet the energy demand during T cell activation (Figure 11d). Collectively, these findings demonstrate that T cell activation regulates metabolic phenotype by maintaining oxidative phosphorylation and increasing the glycolysis, which dominant in activated T cells for energy production to meet higher energy demand following cell activation.

## Discussion

The use of , a centric marine diatom with relatively well-conserved pore geometry and size (∼5–10 μm diameter frustules, uniform areolar arrays), provides a structurally regular scaffold for antibody conjugation. Nonetheless, as a biologically derived material, some microarchitectural variability in pore spacing, wall thickness, and curvature can exist. While our current study did not quantify these parameters, future work may utilize microCT imaging, high-resolution SEM, or automated morphometric tools to assess and control batch uniformity. Such structural profiling would be valuable in ensuring reproducibility and tuning performance in functional applications.

To our knowledge, this study is the first to document the creation of biomimetic artificial APCs utilizing diatoms modified with Ca­(OH)_2_. This innovative approach involves the oxidative polymerization of dopamine, followed by the immobilization of anti-CD3 and anti-CD28 antibodies on the diatom surface. Consequently, this process results in the development of diatom-based aAPCs aimed at modulating adaptive immune responses.

In addition to their natural micro- and nanoarchitecture, diatom exhibits inherent mechanical rigidity, with elastic moduli in the gigapascal range. This stiffness may offer distinct advantages for aAPC applications. Rigid surfaces have been shown to enhance mechanotransduction at the immunological synapse, promoting TCR clustering, cytoskeletal reorganization, and downstream signaling pathways. Unlike deformable hydrogel-based aAPCs that seek to mimic the soft nature of physiological APCs, diatom-based platforms may provide a stable and mechanically supportive interface, particularly under conditions of flow or shear stress where structural durability is critical. Prior studies using rigid platforms such as Dynabeads have successfully activated T cells, suggesting that mechanical compliance is not the only determinant of activation efficiency. The unique combination of bioderived surface complexity and mechanical robustness makes diatoms promising scaffolds for scalable, long-term use. Nevertheless, future iterations may explore hybrid materials that incorporate compliant coatings or surface modifications to further tune mechanobiological interactions without compromising structural integrity.

Ca­(OH)_2_-modified diatoms were synthesized as described in our previous study.^17^ For dopamine (DOPA) polymerization of Ca­(OH)_2_-modified diatoms, we optimized the dopamine coating to achieve stable amine functionalization on Ca­(OH)_2_-modified diatoms by varying the ratios of diatoms to dopamine and adjusting incubation times. These conditions were optimized to maximize free amine availability on the diatom surface, which is critical for subsequent antibody attachment. Dopamine was chosen because of its ability to undergo oxidative polymerization, forming a thin polydopamine (polyDOPA) layer that can strongly adhere to a variety of surfaces, including inorganic materials such as calcium-modified diatoms. This layer is rich in catechol and amine groups, which facilitate the attachment of biomolecules through covalent or electrostatic interactions,^17^ enhancing the functionality of diatom aAPCs in immune cell engagement. Our findings suggest that a 1:2 diatom-to-dopamine ratio with an 8-h incubation time results in a stable and functional amine layer on calcium-modified diatoms, suitable for further antibody immobilization. This result aligns with previous studies in which dopamine coatings effectively introduced amine groups onto various biomaterial surfaces, enhancing their bioconjugation potential for biomedical applications.^18^

A critical limitation in the development of aAPCs is the maintenance of the stability and functionality of the attached biomolecules over time. Silica-based diatoms are effective^19^ but often suffer from rapid antibody degradation due to environmental instability. In contrast, dopamine-coated calcium-modified diatoms demonstrated enhanced stability, as evidenced by the minimal degradation of diatoms across multiple days. A study reported that in situ polydopamine-based coating on diatom surfaces is resistant to degradation under detergent and acid treatments.^20^ In addition, Ca^2+^ ions in the diatom likely provide a stabilizing effect, acting as a cross-linking agent and reinforcing the polydopamine matrix through Ca^2+^-catechol coordination. This interaction reduces oxidative degradation and maintains structural integrity over time.^21^ When Jurkat cells were treated with dopamine-coated Ca­(OH)_2_-modified diatoms, we observed higher cell viability and metabolic activity, suggesting no cytotoxic effect of diatoms on cells. A previous study also reported that dopamine-coated diatoms exhibit low toxicity to Jurkat cells, with over 90% cell viability maintained even at high concentrations.^22^ One limitation of diatoms is that, the higher viability observed in the diatom group may be due to physical or optical interference from the diatoms with the resazurin assay signal at later time points. Therefore, it was higher in comparison to Jurkat cells alone. To this extent, we employed cell density and flow cytometry as more reliable measures for viability studies.

This functionalization strategy represents a critical step toward developing effective diatom-based aAPCs, as it allows for the reliable attachment of costimulatory molecules necessary for T cell activation, such as antibodies. Herein, we successfully synthesized diatom-based aAPCs by immobilizing anti-CD3 and anti-CD28 antibodies on dopamine-coated Ca­(OH)_2_-modified diatoms. This was achieved by quantifying and visualizing fluorophore-conjugated antibodies on the diatom surface while maintaining its unique three-dimensional (3D) fultoportulae microstructure. Polydopamine is known for its biocompatibility and capacity to enhance cell-material interactions through its catechol and amine functional groups, which interact with the amine and thiol groups of antibodies to facilitate antibody immobilization.^23^ Our results are consistent with those of other studies that employed dopamine’s adhesive properties to functionalize surfaces with biomolecules such as CD34 through oxidative polymerization.^24^ While fluorescence microscopy allowed qualitative confirmation of antibody conjugation, quantification using image-based analysis was confounded by the irregular morphology and depth of the diatom particles. Future studies should incorporate fluorescence calibration or flow cytometry (FACS)-compatible microstructures for quantitative assessment of antibody loading and surface coverage.

T cell activation is a hallmark of adaptive immune responses. T cell activation involves a coordinated sequence of antigen recognition, costimulatory signaling, and intracellular signaling, with calcium playing a pivotal role in modulating these processes.^25^ We confirmed the proof-of-concept functionality of diatom-based aAPCs through the activation of Jurkat cells and further translation studies on primary CD4^+^ T cells, the model for T cell behavior. We found that anti-CD3 and anti-CD28 antibodies immobilized on dopamine-coated, calcium-modified diatoms enhanced the viability of Jurkat cells. This aligns with previous findings showing that anti-CD3/CD28-coated beads are highly effective in promoting the expansion of CD4^+^ human T cell populations.^26^

Dopamine coatings on diatoms improve biocompatibility by masking surface antigens that may trigger immune responses.^27^ We observed increased interactions between diatom-based aAPCs and Jurkat cells, with evidence of stable immunological synapses forming shortly thereafter. This is due to the adhesion of T cells (receptors expressing CD3 and CD28) and diatom-based aAPC that exhibit anti-CD3 and anti-CD28. The immunological synapse is a critical structure for T cell activation, facilitating direct signaling through the TCR and costimulatory molecules.^28^ Our results are supported by a previous study that demonstrated that beads coated with anti-CD3 and anti-CD28 promoted human T cell activation and expansion in vitro.^29^ However, the specificity of T cell activation via TCR/CD28 engagement could be further informed by incorporating the blocking antibodies and isotype controls.

Intracellular calcium plays a crucial role in T cell activation. By incorporating calcium into the diatom structure and functionalizing the surface with dopamine, we created a robust scaffold that not only supports stable antibody attachment but also enhances T cell activation through costimulatory signaling. The higher stability of Ca­(OH)_2_-modified diatoms combined with dopamine coatings is particularly advantageous for aAPC applications, where prolonged exposure to physiological environments is required for immune cell interactions.^30^ Using the calcium-sensitive dye Fluo-4, we further examined the intracellular calcium flux as a marker of T cell activation. When T cells interacted with antibody-coated, calcium-modified diatoms, we observed a substantial increase in Fluo-4 fluorescence, indicating an influx of calcium ions at early phase of T cell activation. Our study provides the added benefit of calcium-modified diatom platforms that enhance cell signaling during T cell engagement compared to traditional silica scaffolds.^31^ This sustained calcium flux is consistent with effective TCR engagement and costimulatory signaling. Upon TCR engagement, calcium is released from the endoplasmic reticulum, triggering store-operated calcium entry (SOCE) and activating key transcription factors, such as nuclear factor of activated T cells (NFAT),^28^ which are necessary for driving further downstream activation processes.

To further verify the translational efficacy of calcium-modified, antibody-functionalized diatoms as aAPCs, we conducted a detailed analysis of CD4^+^ T cell activation by assessing surface marker expression, proliferation, and cell metabolism. Diatom-based aAPCs demonstrated upregulation of CD69 expression early on while facilitating stable CD25 expression in CD4^+^ T cells at a later time point. These markers, particularly CD69 and CD25, are critical indicators of the T cell activation status. CD69 is an early activation marker that appears within hours of TCR engagement, whereas CD25 (the α chain of the IL-2 receptor) is a late activation marker essential for prolonged cell activation and proliferation.^32^ CD69 is immediately detected early after T cell activation and declines rapidly after 4–6 h. In activated lymphocytes, CD69 suppresses sphingosine 1-phosphate receptor 1 (S1P1) signaling by facilitating receptor internalization and degradation. First, it enhances the JAK3/pSTAT5 pathway, which counteracts STAT3-driven IL-17 expression and supports regulatory T cell (Treg) development.^33^ Sustained expression of CD25 is regulated by IL-2-induced STAT5 (Signal Transducer and Activator of Transcription 5) signaling, which reaches its peak approximately 48 h after activation.^34^

Proliferation study revealed a progressive increase in proliferation of primary T cells (CD4^+^ PBMCs) in the presence of aAPC-derived diatoms starting from day 1, with greater division heterogeneity by day 5. CFSE proliferation data involves generation-resolved peaks, where each subsequent T cell division is visible as a distinct peak of lower fluorescence intensity. However, due to low proliferation rates under serum-limited and cytokine-free conditions, limited duration of culture (typically <72 h), and use of bulk CD4^+^ PBMCs, the number of cell divisions was insufficient to resolve clear multipeak distributions. Thus, we only observed a general left-shift signals that reflect dilution of CFSE, indicating cell proliferation.

Mitochondrial respiration and glycolysis play vital roles in T cell activation by supplying the essential energy and metabolic intermediates required for their function and proliferation. T cells interacting with the functionalized diatoms exhibited robust proliferation, which is consistent with reports that costimulatory signaling, such as that provided by CD3 and CD28, is critical for both initiating T cell activation and supporting cell cycle progression for clonal expansion.^35,36^ Our study revealed that CD4^+^ T cell activation induced by diatom-based aAPCs maintained their mitochondrial respiration and oxidative phosphorylation, indicating ATP production via aerobic metabolism. In contrast, we observed increased glycolysis, suggesting robust anaerobic ATP generation to meet elevated energy demands before cell activation. Our study indicates that T cells rely on glycolysis for energy. Activation of the TCR, along with costimulatory signals, enhances nutrient uptake and metabolic activity to support elevated energy demands. To sustain this heightened metabolic requirement, activated T cells upregulate glycolysis, mitochondrial biogenesis, and oxidative phosphorylation.^37^

Although this study focused on CD4^+^ T cells, we acknowledge the critical role of CD8^+^ cytotoxic T lymphocytes in cancer immunotherapy. Our Diatom-aAPC platform was designed to engage the TCR/CD28 complex, a mechanism shared by both CD4^+^ and CD8^+^ subsets. Future studies should investigate subset-specific responses, particularly to assess the capacity of this platform to stimulate antigen-specific CD8^+^ cytotoxic responses.

While CD25 upregulation is widely used as an indicator of sustained T cell activation, it is also a canonical marker of regulatory T cells (Tregs), particularly when coexpressed with FOXP3. In this study, we did not assess FOXP3 expression and therefore cannot exclude the possibility of Treg induction. However, the observed expression kinetics of CD69 and CD25, along with intracellular calcium flux and enhanced proliferation, are more consistent with an effector T cell activation phenotype. Nevertheless, we acknowledge this as a limitation. Future studies should incorporate Treg-specific markers such as FOXP3 to further characterize the functional phenotype of CD25^+^ T cells and confirm the suitability of Diatom-aAPCs for pro-inflammatory applications such as cancer immunotherapy.

Another critical factor in artificial antigen-presenting cell (aAPC) design is the ratio and density of immobilized costimulatory antibodies. In this study, we employed an equimolar (1:1) ratio of anti-CD3 and anti-CD28 antibodies during the conjugation process, based on prior studies that demonstrated enhanced T cell activation with this stoichiometry on bead-based platforms such as Dynabeads (Zappasodi et al.). While our findings confirm stable antibody immobilization through fluorescence microscopy, we did not quantify surface density per particle. This remains a limitation of our current study. Future investigations will focus on optimizing antibody loading and spatial orientation using quantitative methods such as fluorescence calibration or ELISA-based surface quantification to standardize and enhance the immunostimulatory capacity of the Diatom-aAPC system.

While this study establishes the immunostimulatory potential of diatom-aAPCs through T cell activation, proliferation, and metabolic reprogramming, functional assessments in disease-relevant models remain a key next step. Future studies will incorporate coculture assays with tumor cells and explore CAR-T cell expansion and cytotoxicity profiling to validate the platform’s therapeutic relevance in oncology settings. Table 1 summarizes the comparison of diatom-based aAPCs with previous aAPC strategies, such as polymeric beads and lipid-based vesicles, highlighting the novelty of diatoms as a potential platform for immunotherapeutic applications.

**Table 1:** Comparison of Diatom-Based aAPCs with Conventional aAPC Strategies, Highlighting the Key Features and Novel Aspects of Diatom-Based System

feature	diatom-based aAPCs (Novel)	polymeric beads	lipid-based vesicles
material	natural biosilica (diatom frustules)	synthetic polymers (e.g., dynabeads, polystyrene, PLGA)	lipid bilayers (e.g., liposomes)
surface area	ultrahigh due to porous nanostructure	moderate	high
chemical functionalization	surface-rich with hydroxyl groups for stable bioconjugation	easy to modify with antibodies/ligands	incorporate MHC/antibodies in lipid layers
3D structure	intrinsically hierarchical 3D nano/microtopography	microspheres	vesicles
stiffness	rigid	solid; tunable	soft; tunable
biomechanical functionality	rigid surface providing stable mechanical support; enhances mechanotransduction, promotes TCR clustering and cytoskeletal reorganization	solid surface designed to mimic the biomechanical environment of natural APCs	soft surface mimicking the soft nature of physiological APC membranes
cell–material interaction	mimics natural APC morphology; promotes cell engagement through TCR	limited by smooth surface	mimic cell membranes
biocompatibility	excellent; biodegradable and inert biosilica	good	good
scalability	scalable and eco-friendly from diatom cultivation	commercially available	lab-scale, sensitive
novelty	new platform offering unique topographical and chemical modification	established	biomimetic

Overall, we successfully demonstrated the synthesis and characterization of diatom-based aAPCs using a combination of calcium modification and dopamine-mediated antibody immobilization. The ability of diatom-based aAPCs to serve as a stable platform to induce T cell activation triggers essential downstream pathways necessary for T cell proliferation and mitochondrial functions, making it a vital component in modulating immune responses. These findings underscore the potential of diatoms as aAPCs for immunotherapeutic applications, particularly in cancer treatment.

## Conclusions

This study presents a novel approach for engineering artificial antigen-presenting cells (aAPCs) using calcium-modified diatoms as a natural, bioinspired platform for targeted T cell activation. By enhancing surface functionalization through amine polymerization, we successfully immobilized key immunomodulatory proteins (anti-CD3 and anti-CD28) for the synthesis of diatom-based aAPCs. We demonstrated that these aAPCs effectively engaged with T cells, forming immunological synapses, which are critical structures for T cell activation. Through costimulatory signals, diatom-based aAPCs demonstrated upregulation of both early and late activation markers for sustained T cell activation, which enhanced cell proliferation. Metabolic profiling revealed higher glycolysis than mitochondrial respiration to meet the energy demands of activated T cells, indicating a robust and adaptive immunometabolic response. Our findings suggest that diatom-based aAPCs have significant potential for application in targeted cancer immunotherapy.

## Methods

### Materials and Reagents

Sodium metasilicate nonhydrate, 7-fluorobenzo-2-oxa-1,3-diazole-4-sulfonic acid ammonium (SBDF), *N*-(3-(dimethylamino)­propyl)-*N*-ethylcarbodiimide (EDC), *N*-Hydroxysuccinimide (NHS), dimethyl sulfoxide, methanol, buffer reagents, artificial seawater reagents, and TNBSA, were purchased from Sigma-Aldrich (Ireland). Lysosensor Yellow/blue DND-160 (PDMPO), alamarBlue, LIVE/DEAD viability, CellTrace CFSE cell proliferation kit, Fluo-4 AM, Dynabeads and TRIzol reagent were purchased from Invitrogen (Ireland). Random primers, reverse transcriptase, 5× Reaction Buffer, 25 mM MgCl_2_, dNTP mix, and recombinant RNasin ribonuclease were purchased from Bioline (Dublin, Ireland). Monoclonal anti-CD3 (clone PC3/188A), anti-CD28 (clone CD28.2), anti-CD69 (clone FN50), anti-CD4 (clone H-370) and anti-CD25 were purchased from Thermo Fisher Scientific (Ireland). Seahorse XF Mito stress test kit was purchased from Agilent.

### Fabrication of Ca­(OH)_2_-Modified Diatoms

 was selected as the model diatom, as described in our previous study.^38^ was well-characterized for its genome. It exhibited hierarchical frustule microstructure with the large surface area that has higher availability of reactive hydroxyl functional groups allow for chemical modification.^5^ Diatoms (microalgae with intricate silica-based cell walls) were cleaned and prepared for calcium modification following our established (Ca­(OH)_2_)-modified diatom.^17^ Briefly, the native silica structure of the diatoms was modified using a calcium deposition technique, replacing silica with calcium to enhance biocompatibility and stability. Diatoms were exposed to a solution of 240 μM calcium hydroxide as a culture medium additive under a controlled pH range of seawater from 7.00 to 9.00 and temperature conditions to promote the formation of a calcium-based matrix within the diatom structure.

### Dopamine Coating for Amine Functionalization on Ca­(OH)_2_-Modified Diatom Surface

To enable biomolecule attachment, calcium-modified diatoms were coated with dopamine, a versatile polymer that adheres to various surfaces. Dopamine undergoes oxidative polymerization under mildly basic conditions, forming a polydopamine layer rich in catechol and amine groups, which provides reactive sites for further antibody functionalization.^17^ Briefly, Ca­(OH)_2_-modified diatoms were incubated in dopamine hydrochloride solution (1 mg/mL, pH 8.5) at three different weight-to-weight (w/w) diatom-to-dopamine ratios (1:1, 1:2, and 1:3) for varying times (1, 2, 8, and 12 h). The conditions were optimized to maximize the availability of free amine groups. Following incubation, the dopamine-coated diatoms were thoroughly washed with phosphate-buffered saline (PBS) to remove any unbound dopamine. As a positive control, (3-aminopropyl)­triethoxysilane (APS) was used as an alternative surface amine functionalization agent. While this was initially used for comparative purposes during early stage optimization, it was not pursued in the final protocol.

The presence of free amine groups on the dopamine-coated surface was confirmed using the 2,4,6-trinitrobenzenesulfonic acid (TNBSA) assay, a colorimetric assay specific for primary amine groups. Briefly, dopamine-coated Ca­(OH)_2_-modified diatoms were dissolved in 1 mL of reaction buffer containing 0.1 M sodium bicarbonate at pH 8.5. Each sample (200 μL) was then mixed with 100 μL of 0.01% TNBSA solution and incubated at 37 °C for 2 h. Subsequently, 100 μL of 10% SDS solution and 50 μL of 1 N HCl were added to each 100 μL of the reaction mixture. All the samples were prepared in triplicate. The concentration of free amine groups in the samples was quantified by measuring the ultraviolet (UV) absorbance of the supernatant at 335 nm using a Varioskan Flash microplate reader (Thermo Fisher Scientific, Finland) and compared with a glycine-based standard curve.^39^

The presence of primary amine groups (−NH_2_) in the dopamine-coated Ca­(OH)_2_-modified diatoms was analyzed using Fourier transform infrared (FTIR) spectroscopy. Samples prepared at varying dopamine incubation times (1, 2, 8, and 12 h) were vacuum-dried and subsequently examined using an FTIR spectrometer (Varian 660-IR, Agilent Technologies, Ireland), with a blank KBr pellet serving as the background reference. The –NH_2_ groups were indicated by peaks at 3300 cm^–1^ and 1650 cm^–1^.

### Antibody Immobilization as a Final Step for the Synthesis of Diatom-Based aAPCs

The functionalized diatoms (dopamine-coated Ca­(OH)_2_-modified diatoms) were further modified by immobilizing monoclonal anti-CD3 and anti-CD28, two essential costimulatory molecules required for T cell activation. Anti-CD3 and anti-CD28 antibodies were conjugated to the amine-rich polydopamine surface on diatoms using EDC/NHS chemistry, which activates carboxyl groups on antibodies, allowing them to form stable covalent bonds with amine groups on the diatom surface.^40^ Anti-CD3 was also conjugated with phycoerythrin (PE), while anti-CD28 was conjugated with Fluorescein isothiocyanate (FITC), enabling visualization under fluorescence microscopy to verify successful antibody immobilization. Anti-CD3 (PE-labeled) and anti-CD28 (FITC-labeled) antibodies were added to the dopamine-coated diatoms at equimolar concentrations (1 μg/mL each) during EDC/NHS coupling to target a 1:1 surface presentation. This ratio was selected based on prior findings showing that equimolar CD3/CD28 antibody presentation on aAPCs enhances T cell activation and proliferation (Zappasodi et al.). While the precise surface density was not quantified, conjugation was qualitatively verified via dual-channel fluorescence microscopy. Briefly, the functionalized diatoms were incubated with antibodies in a solution containing 0.2 M EDC and 0.05 M NHS for 2 h at room temperature. Excess antibodies were removed by extensive washing with PBS for further characterization. The success of antibody attachment was determined using an inverted fluorescence microscope (Olympus IX81; Olympus Optical Co. Ltd.). The PE-labeled anti-CD3 and FITC-labeled anti-CD28 on diatoms were analyzed for the mean fluorescence intensity (Au) using ImageJ version 1.48 (National Institutes of Health).

### Viability of Jurkat Cells

The cytotoxicity of diatom-based aAPCs was investigated using Jurkat cells. Briefly, 5 × 10^3^ cells were seeded in four well chambers and allowed to grow for 5 days in the presence of diatom-based aAPCs at 37 °C in a humidified atmosphere of 5% CO_2_. The morphology and viability of Jurkat cells in the presence of dopamine-coated Ca­(OH)_2_-modified diatoms were stained by LIVE/DEAD kit. The cultures were undisturbed for 10 min prior to imaging. During this time, many cells passively settled onto the glass-bottom imaging surface. Imaging was performed using fluorescence microscopy, and the *Z*-plane was selected to capture the maximally populated focal layer. The metabolic activity of Jurkat cells was also determined using the alamarBlue assay. All experiments were performed in triplicate. The density of Jurkat cells was determined using a hemacytometer.

### Isolation of CD4^+^ Peripheral Blood Mononuclear Cells

CD4^+^ T cells were isolated from human peripheral blood mononuclear cells (PBMCs) using the Pan T Cell Isolation Kit II (Miltenyi Biotec) employing a negative selection strategy. This method removed non-T cells (for example, B cells, NK cells, monocytes, dendritic cells, granulocytes, and erythrocytes) via magnetic with antibody-conjugated beads. The unlabeled, untouched T cells remained in suspension and were collected for downstream applications. This approach ensures that the isolated T cells are not activated during the isolation process, maintaining their native state and functional integrity, which is crucial for accurate activation and expansion.

### Surface Morphology by Scanning Electron Microscopy

Cleaned diatom frustules (with and without Jurkat cells) suspended in methanol were air-dried on carbon stubs and subsequently coated with gold. Briefly, Jurkat–diatom complexes were fixed in 2.5% glutaraldehyde in PBS for 30 min at room temperature, followed by washing in PBS for three times. Samples were dehydrated through a graded ethanol series (30, 50, 70, 90, 100%) for 10 min each step. Final drying was done via hexamethyldisilazane (HMDS) treatment to preserve cell morphology and interactions. Samples were sputter-coated with gold/palladium (80:20) using a Quorum Q150R ES Sputter Coater under the following conditions: current of 20 mA, coating time of 60 seconds, chamber pressure of 0.05 mbar and target distance of 5 cm. The surface morphology of gold-coated samples was examined using a Hitachi S-4700 SEM equipped with INCA software (Oxford Instruments). Analyses were conducted on the exposed valve faces of the frustules.

### Degradation Study

Equal amounts of diatoms were prepared for each group, and their initial dry weights (*W*_do_) were recorded before incubation in PBS. Specifically, 2.0 mg of dry diatom material was used per condition. Samples were incubated in 1 mL PBS at 37 °C and collected at designated time points (days 3, 7, 14, 28, 56, and 112). Diatoms were then washed with deionized water, followed by drying at 60 °C for 1 h in a vacuum drying oven. The final dry weights (*W*_dt_) were determined using an analytical microbalance (Mettler Toledo XS105, readability 0.01 mg). All measurements were performed in triplicate and the mean ± SD was reported. Degradation was evaluated by calculating the percentage of weight loss over time using the following formula

$\mathrm{dry}\;\mathrm{weight}\;\mathrm{loss}\;\left(\%\right)=\frac{W_{\mathrm{do}}-W_{\mathrm{dt}}}{W_{\mathrm{do}}}\times 100\%$

### T Cell Activation Markers

To assess T cell activation markers, Jurkat and primary CD4+ T cells were incubated with diatom-based aAPCs. Both early and late activation markers (CD69 and CD25, respectively) were measured using RT-qPCR and flow cytometry.

On day 5 of incubation with diatom-based aAPCs, RNA was extracted from CD4^+^ T cells cultured in monolayers using TRIzol reagent (Invitrogen) in combination with the miRNeasy Mini Kit (Qiagen), according to the manufacturer’s instructions. A total RNA concentration of 100 ng/μL was used for reverse transcription with random primers (Bioline), followed by cDNA synthesis using reverse transcriptase (Bioline) in a 20 μL reaction volume, performed using a PTC-200 DNA Engine Thermal Cycler (MJ Research, Inc.). cDNA was amplified using SYBR Green PCR Master Mix (Promega) with primers specific for CD69 and CD25. Quantitative PCR was performed in triplicate using the StepOnePlus Real-Time PCR System (Applied Biosystems). Gene expression data were analyzed using the 2^–ΔΔCt^ method^41^ and expressed as fold change relative to HPRT expression.

After 5 days of incubation with diatom-based aAPCs, T cells were stained with fluorophore-conjugated antibodies against CD69 and CD25, and fluorescence was measured using a BD FACSCanto flow cytometer. This provided quantitative data on the percentage of T cells expressing activation markers, allowing for comparison across different functionalization conditions.

### Calcium Imaging to Assess Intracellular Signaling

At 2 h postincubation with diatom-based aAPCs, intracellular calcium influx, a hallmark of T cell activation, was measured using Fluo-4, a calcium-sensitive dye that fluoresces upon binding to free calcium ions. CD4^+^ T cells were loaded with Fluo-4 AM dye in a calcium-containing buffer for 30 min at 37 °C, washed, and incubated with diatom-based aAPCs for 5 days. Changes in fluorescence intensity were observed using fluorescence microscopy, and calcium influx was measured to assess T cell activation. A higher fluorescence intensity indicates a strong activation response, reflecting efficient TCR engagement and costimulation.

The binding between primary human T cells (CD4^+^ peripheral blood mononuclear cells (PBMCs)) and aAPCs-derived diatoms in calcium starve-, calcium- and silica-feed conditions was further informed. Calcium feed when the diatoms were pretreated with Ca­(OH)_2_ to enhance surface amine reactivity prior to dopamine polymerization. Calcium starve condition when the diatoms were not exposed to Ca­(OH)_2_, representing an unmodified silica surface before dopamine coating. Silica feed was referred to unmodified, native diatom silica used directly without surface chemistry modification.

### T Cell Proliferation Assay

T cell proliferation was assessed using CFSE staining, a commonly used method to monitor cell division by measuring dye dilution over multiple generations. T cells were labeled with CFSE and incubated with diatom-based aAPCs for over 5 days, simulating prolonged aAPC interaction. Flow cytometry was used to measure the CFSE intensity at various time points, tracking the reduction in fluorescence with each cell division cycle.

### Seahorse Extracellular Flux Analysis for Metabolic Activation

Seahorse extracellular flux analysis was performed to measure changes in metabolic activity, particularly in glycolysis and oxidative phosphorylation. After 5 days in culture, CD4^+^ T cells incubated with diatom-based aAPCs were placed in a Seahorse XF Analyzer, and the OCR and ECAR were measured following the manufacturer’s protocol.^42^ These metabolic readouts served as indicators of the activation state, with increased OCR and ECAR suggesting high metabolic activity consistent with T cell activation.

### Statistical Analysis

Statistical analyses were performed using GraphPad Prism version 9.00 software. Data were compared using one-way analysis of variance (ANOVA), and multiple pairwise comparisons were performed using the Bonferroni *post hoc* test for amine concentration, fluorescence intensity, and percentage interaction. Data were analyzed using two-way ANOVA for degradation study, metabolic activity, cell density, gene expression analysis, OCR, and ECAR. Statistical significance was set at *P* < 0.05.

## Supplementary Material





## Notes

T cell interaction with diatom-based aAPCs (Video S1) (MP4)
Isolation of CD4^+^ primary T cells from PBMCs (PDF)
