# Supplementary material for: Diatom-Based Artificial Antigen-Presenting Cells: A Novel Approach for Adaptive Immune Modulation
Source: ACS Appl Mater Interfaces. 2025 Jun 18;17(26):37718–34. doi: 10.1021/acsami.5c07766 (PMC12232282; doi:10.1021/acsami.5c07766)
Supplement: Supplementary file 2 [file am5c07766_si_002.pdf]

Diatom–based Artificial Antigen-Presenting Cells: A Novel Approach for Adaptive Immune Modulation

Asrizal Abdul Rahman[1], Isma Liza Mohd Isa[1,2,3], Manus J. Biggs[1], Abhay Pandit[1*]

[1]CÚRAM Research Ireland Centre for Medical Devices, University of Galway, H91 W2TY Galway, Ireland

[2]Pharmacology and Therapeutics, School of Medicine, University of Galway, H91 TK33 T Galway, Ireland

[3]Department of Anatomy, Faculty of Medicine, Universiti Kebangsaan Malaysia, 56000 Kuala Lumpur, Malaysia

*corresponding email: abhay.pandit@universityofgalway.ie

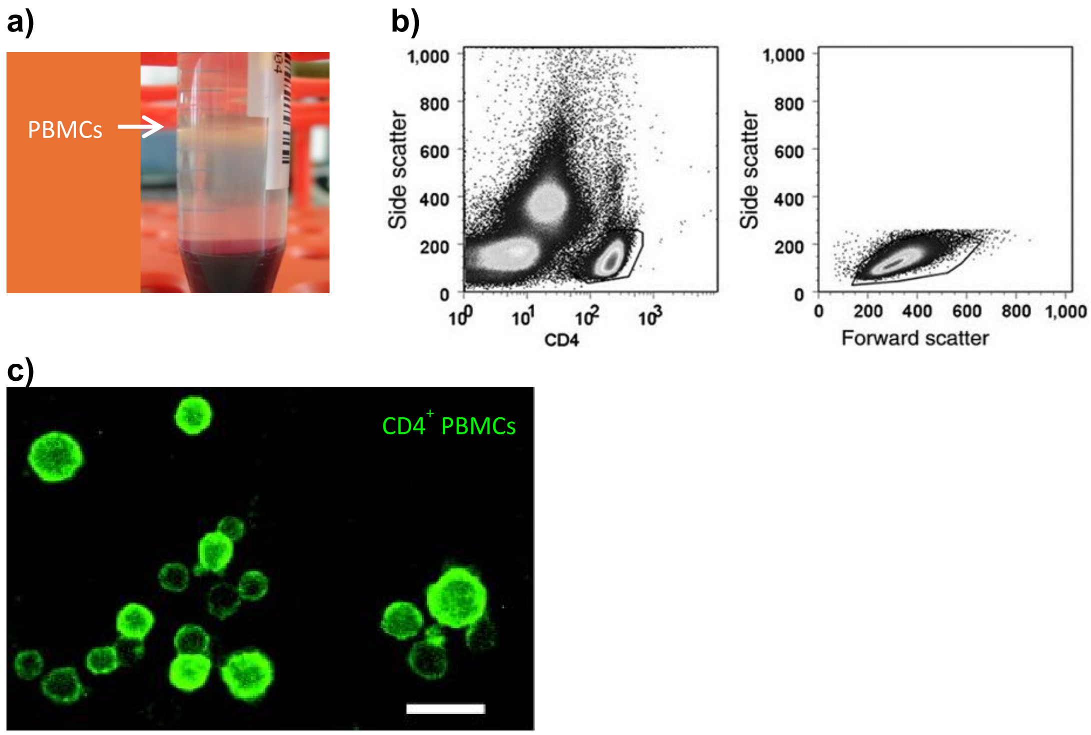

Supplementary Figure S1. Isolation of $CD4^+$ primary T cells from PBMCs. **a)** Layer of PBMCs, which the cells were isolated from peripheral blood using Ficoll density gradient separation. **b)** Magnetic sorting using flow cytometry revealed the purity of untouched $CD4^+$ PBMCs, indicating the primary T cells have been successfully isolated. **c)** Fluorescence microscopy determined the morphology of fluo-4 labelled-$CD4^+$ PBMCs. n = 3. Scale bars represent 10 μm for (c).
